# Development of Loop-Mediated Isothermal Amplification Combined with Lateral Flow Dipstick Assay for a Rapid and Sensitive Detection of Cystic Echinococcosis in Livestock in Kenya

**DOI:** 10.1155/2022/4928009

**Published:** 2022-02-27

**Authors:** Nasser Arreh Badoul, John Kagira, Florence Ng'ong'a, Hunduma Dinka

**Affiliations:** ^1^Department of Molecular Biology and Biotechnology, Pan African University Institute for Basic Sciences, Technology and Innovation, Juja, Kenya; ^2^Department of Animal Sciences, School of Natural Resources and Animal Sciences, Jomo Kenyatta University of Agriculture and Technology, Juja, Kenya; ^3^Department of Biochemistry, School of Biomedical Sciences, Jomo Kenyatta University of Agriculture and Technology, Juja, Kenya; ^4^Department of Applied Biology, School of Applied Natural Sciences, Adama Science and Technology University, Adama, Ethiopia

## Abstract

**Background:**

Cystic echinococcosis is a zoonotic disease caused by the metacestode stage of *Echinococcus granulosus* and occurs worldwide, causing considerable economic losses and public health problems. The currently available methods for the diagnosis of animal hydatidosis are time-consuming and require well-equipped laboratories which make them incompatible with testing in resource-poor settings. This study developed and evaluated a rapid, more sensitive, and specific loop-mediated isothermal amplification combined with a lateral flow dipstick assay for the rapid and sensitive detection of cystic echinococcosis.

**Results:**

In this study, a specific primer set and FITC-labeled probe targeting the conserved region of the NADH-1 gene were designed. The LAMP reaction was performed at 60°C for 40 minutes, and the amplification products were successfully visualized by LFD strips. The analytical sensitivity of LAMP-LFD was determined using 10-fold serial dilutions of *E. granulosus* DNA. The minimal concentration detected was 10 fg/*μ*l, and no cross-reactivity was observed with DNA extracted from *Taenia solium*, *Taenia saginata,* and *Fasciola hepatica*. The ability of the developed LAMP-LFD assay to detect cystic echinococcosis was further evaluated with 62 cyst samples from slaughtered cattle in Juja Abattoir, Kiambu County, Kenya. The LAMP-LFD was able to detect 59/62 (95.2%, 95% CI 0.87–0.98) as positive samples of *E. granulosus* compared to 53/62 (85.5%, 95% CI 0.75–0.92) by nested PCR assay.

**Conclusion:**

Our results indicated that the developed LAMP-LFD technique was more sensitive than the nested PCR assay, rapid, and easy to perform with a simple visual detection of products. Therefore, it could be an important point-of-care diagnostic tool for cystic echinococcosis.

## 1. Background

Cystic echinococcosis (CE) is a neglected parasitic disease caused by the larval stage of the tapeworm *Echinococcus granulosus* (sensu lato), and infections involve both human and ungulate animals [1]. The disease is worldwide spread and remains a major public health concern in many low-income sub-Saharan countries, especially where livestock are raised under free-range conditions as a food source [2]. CE has an economic implication because any organ found to be infected with *E. granulosus* during meat inspection is condemned and judged to be unfit for human consumption, and this may lead to food insecurity due to the loss of meat for human consumption [2, 3]. The life cycle of *E. granulosus* involves two animal hosts, the dog being the main host for the definitive life cycle stage, whereas the hydatid cyst (larval stage) is found in many warm-blooded animals including cattle, sheep, goats, and camels, as well as in humans [4]. Effective diagnosis of livestock hydatidosis is vital because ingestion of condemned infected organs by dogs creates a pool of reservoirs and enables continuous spread to humans and cattle [5].

Unfortunately, the conventional diagnostic method based on visual examination during meat inspection is not sufficient and has a relatively low diagnostic sensitivity and may not detect 15.4% of cases, as the cysts are sometimes not clearly visible, especially in the early developmental period and in degradative cysts [6, 7].

Several serological examination techniques have been developed for the detection of *E. granulosus.* However, these methods have low sensitivity and cross-reactivity is likely to outcome with other taeniid cestodes [6, 8].

Molecular-based methods are more useful and reliable and play an important role in the diagnosis and genotyping of *E. granulosus* infection in animals [9, 10]. Most of the molecular diagnosis and genotyping of *E. granulosus* have relied on the use of mitochondrial *E. granulosus* DNA (mtDNA) as the target region since it is a plasmid-like multicopy nonnuclear DNA that contains a conserved sequence of the NADH-1 gene and is easy to extract using the alkaline and boiling methods [11, 12].

Agarose gel-based PCR assays [11, 12] and real-time PCR (RT-PCR) techniques [13, 14] have been developed and adopted for molecular detection and genotyping of *E. granulosus* infection in livestock. But these techniques are laboratory-based and costly, thus unsuitable and inapplicable for most low-income countries with low-resource settings where cystic echinococcosis is endemic [15]. The lack of affordable, easy-to-use, sensitive, and specific molecular diagnostic tools for the detection of *E. granulosus* infection at the slaughterhouse level hinders the prevention and control of CE in areas of endemicity [16].

Several isothermal nucleic acid amplification methods that do not rely on the thermocycling process have been developed. Loop-mediated isothermal amplification (LAMP) was developed [12] as a rapid, simple, and highly sensitive and specific molecular diagnostic technique when compared to PCR-based methods [17]. The LAMP assay amplifies DNA at a constant temperature of 60–65°C. Based on these features, the LAMP technology has gained momentum for nucleic acid amplification and has been widely used in various fields including biomedical, agriculture, veterinary, aquaculture, food, and environmental sciences [18]. The LAMP technique has been evaluated as a molecular diagnostic tool for a variety of pathogenic microorganisms including *E. granulosus* [19–21].

There are various detection methods for LAMP reaction, where real-time detection of DNA amplification can be monitored by measuring turbidity in real-turbidimeter devices or using fluorescence, but this method requires special and sophisticated equipment. Agarose gel electrophoresis is the most common method for analyzing LAMP results [22]. However, an individual LAMP technique may produce cross-amplification (nonspecific products), resulting in false-positive products. As a result, hybridization with a specific DNA probe or restriction endonuclease digestion should be used to verify the LAMP amplicons [23]. Although the colorimetric indicators such as SYBR green dye are highly sensitive and time-effective with simple visual detection of products, their level of specificity is low and may produce false positives because they can bind any double-stranded DNA including primer dimers [24].

Recently, a commercially available lateral flow dipstick (LFD) test has been developed for detecting amplified DNA by the LAMP assay [23]. The use of the LFD assay is a major factor contributing to the improvement of the specificity of the LAMP assay because it utilizes a specific hybridization probe that can only bind to the specific sequence of the biotinylated LAMP products. Due to its high specificity, less time consumption, simplicity, rapidity, cost-effectiveness, and requirement of inexpensive and nonsophisticated equipment, the combination of LAMP with LFD detection makes it an ideal rapid diagnostic tool for use in the field and point-of-care diagnostic applications. This study developed and evaluated the LAMP-LFD assay based on the NADH-1 gene for the detection of *E. granulosus* infection in livestock at slaughterhouses.

## 2. Materials and Methods

### 2.1. Samples and Sampling Method

Reference samples which included *E. granulosus* from cattle (positive control) and seven archived *E. granulosus* samples previously isolated and identified by molecular methods [11] and *Taenia solium, Taenia saginata,* and *Fasciola hepatica* samples for specificity assay were provided by the Centre for Microbiology Research (CMR) Laboratory at Kenya Medical Research Institute (KEMRI), Mbagathi.

Sixty-two hydatid cyst-infected organs collected from slaughtered cattle in Juja Abattoir were used for validation of the LAMP-LFD assay. These samples were collected using the purposive sampling method [25]. The samples were labeled appropriately in sealed bags and then transported to the Molecular Biology and Biotechnology Laboratory at the Pan African University Institute for Basic Sciences, Technology, and Innovation (PAUSTI) in Juja Town, Kenya. The cysts were cut-opened, and fluids were microscopically examined to classify cysts as fertile or infertile with 40X objective and then kept in 70% ethanol at 4°C for molecular downstream processes indicated below.

### 2.2. DNA Extraction from Cysts

The alkaline lysis method was used to extract genomic DNA from protoscoleces and associated germinal layers [4]. Briefly, 20 mg of cyst tissue was crushed in a mortar by pestle, and 25 *μ*L of suspension was prepared into different microtubes containing an equal volume of 0.02 M NaOH and incubated at 99°C for 10 minutes. The lysate was directly used as a DNA template for LAMP and PCR assays.

### 2.3. Design of LAMP Primers and Probe

A set of six primers and probe which include F3/B3 outer primers, FIP/BIP inner primers (FIP and BIP), and LF/LB loop primers were designed targeting the mitochondrial NADH dehydrogenase subunit one (NADH-1) gene of *E. granulosus* published in GenBank in NCBI with accession no. MG672292. The primers were designed by using the online NEB LAMP Primer Design Tool (https://lamp.neb.com/#!/; version 1.0.1, New England Biolab, UK) for screening out good LAMP primer sets.

**Figure 1:**
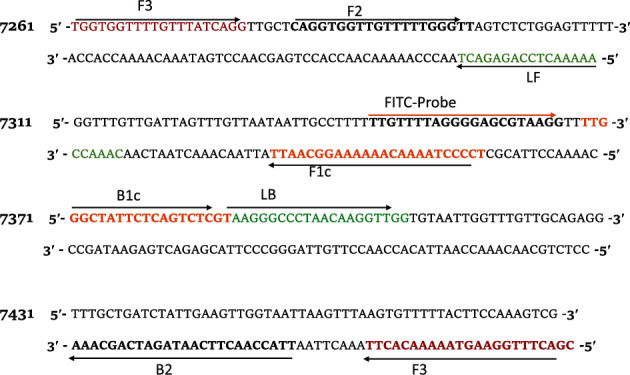
Nucleotide sequence of the NADH-1 gene sequence of *E. granulosus* (GenBank accession number MG672292) used to construct (indicated in bold and color) the outer (F3/B3), inner (FIP/BIP), and loop (LF/LB) primers and probe. The inner primers FIP and BIP comprise the complementary sequences to F1c and B1c, and F2 and B2.

For lateral flow dipstick (LFD) detection, the hybridization probe was manually designed based on the NADH-1 gene of *E. granulosus* between the F1c and B1c primer sequences. The forward inner primer (FIP) was labeled with biotin, and the probe was labeled with fluorescein isothiocyanate (FITC). The primer and probe sequences are shown in Table 1. All the primers were purchased from Macrogen (Seoul, S. Korea) while the probe was obtained from Biomers.net GmbH (Germany). The target nucleotide sequence of the *E. granulosus* NADH-1 gene is presented in Figure 1.

### 2.4. Optimization of LAMP Assay Conditions

Following primer screening, the LAMP assay was conducted using standard LAMP reaction conditions [26]. Briefly, the LAMP assay was performed in 25 µL LAMP amplification reagents containing 2.5 µL of buffer, 1.5 µL MgSO_4_ (100 mM), 3.5 µL dNTP Mix (10 mM), 1 µL of each primer (include outer primers (0.2 µM), inner primers ((1.2 µM), and loop primers (0.4 µM))), and 1 µL Bst DNA Polymerase (8,000 U/mL) (New England Biolab, sMA, USA). The DNA template of 2 µL and sterilized water were added for a final volume of 25 µL. The LAMP reaction was carried out in a Loopamp LA-500 real-time Turbidimeter (SNEAOZO259, EIKEN Chemical Co., LTD, Tokyo, Japan) and adjusted at 63°C for 60 minutes. Nuclease-free molecular grade water was included in the no template control.

For the optimization of the LAMP conditions, different temperatures (60–65°C) and duration times (30–60 minutes with a 10-minute interval) were tested. LAMP reagents optimized using different concentrations of MgSO_4_ (3 mM to 9 mM), dNTPs (0.2–2.0 mM), and LAMP reaction with and without loop primers were tested, and also outer and inner primer ratios were also evaluated. All results were analyzed by gel electrophoresis during the optimization stage.

The LAMP reactions were monitored by analyzing turbidity in a real-time turbidimeter, and the end products were visualized by the naked eye for color change in visible light and under UV light using 2 *μ*L of 1/1000 dilution of SYBR Green 1 dye. Three microliters of the product were analyzed onto 3% gel electrophoresis stained with Gel Red® (Biotium, Inc.) using a gel documentation system (Uvi Tech, UK). The experiments were performed in triplicate.

### 2.5. Lateral Flow Dipstick (LFD) Detection

A biotinylated primer and a probe labeled with FITC at 5′ end were used for the LAMP-LFD detection. Using the optimized LAMP assay conditions, the reaction was set at 63°C for 40 minutes. For hybridization of the probe with the biotinylated LAMP products, 1 L (0.4 µM) of the FITC probe was added and further incubation was done for 5 minutes. Then, 10 µL of the hybridization products was mixed with 90 µL assay buffer, and the lateral flow dipsticks (MGHD2, Milenia HybriDetect 2T, Germany) were then dipped into the mixture, followed by incubation at room temperature for 5 minutes.

For the positive reactions, two visible red lines were visualized, one at the test line (TL) and another at the control line (CL). For negative reactions, only the control line (CL) was observed. The reactions were duplicated using a water bath at 63°C for 40 minutes, followed by reaction inactivation at 80°C for 5 minutes.

### 2.6. Nested PCR Detection

A nested PCR procedure was employed to detect the presence of *E. granulosus* in cyst samples. The first amplification of the NADH dehydrogenase 1 (NADH-1) gene was conducted as described in [27] using primers 5′-TGG AAC TCA GTT TGA GCT TTA CTA-3′ and 5′-ATA TCA AAG TAA CCT GCT ATG CAG-3′ as forward and reverse primers, respectively. A 25 *μ*L reaction mixture was prepared of 12.5 µL of premix Taq buffer (New England Biolabs), 0.5 µL of each primer, 2 µL of genomic DNA, and the final volume of 25 µL of nuclease-free water. Using the Proflex PCR System (Thermo Fisher Scientific, USA), the amplification conditions were as follows: initial denaturation at 95°C for 5 minutes, followed by 40 cycles of denaturation at 95°C for 30 seconds, annealing at 56°C for 45 seconds, elongation at 72°C for 45 seconds, and final elongation at 72°C for 5 minutes.

A second amplification was performed under the same conditions using 2 µL of amplicon produced with first amplification with 5′-TAT TAA AAA TAT TGA GTT TGC GTC-3′ and 5′-TCT TGA AGT TAA CAG CAT CAC GAT-3′ as forward and reverse primers, respectively. The target product size was 1073 bp. The PCR product was resolved on a 1.5% (w/v) agarose gel stained with Gel Red® (Biotium, Inc.) and visualized under UV light (Model UV Doc. HDS UITEC Cambridge, UK). The sizes of the amplicons were estimated by comparing them with a commercial Cleaver CLS-MDNA-1 kb DNA ladder RTU 1151021805 on an agarose gel.

### 2.7. Analytical Sensitivity and Specificity of the LAMP and PCR Assays

The sensitivity of the LAMP and PCR assays was evaluated using different concentrations of *E. granulosus* DNA in descending order by 10-fold serial dilution with DNA elution buffer (10 µL of pure DNA in 90 µL of LFD buffer). 5 µL of DNA was used as template DNA in the LAMP and PCR reactions at 63°C for 40 minutes. The sensitivity test was estimated as the last dilution of each positive reaction. The products were evaluated with LFD strips, 3% agarose gel electrophoresis, and SYBR Green 1 dye. The analytical sensitivity between LAMP and PCR results was compared using 10-fold serial dilutions of the *E. granulosus* DNA template.

In order to determine the LAMP-LFD specificity, 2 µL of *E. granulosus* DNA template was tested with LAMP and the results were compared with the DNA extracted from *T. solium, T. saginata,* and *Fasciola hepatica* worms. The LAMP products were analyzed on the LFD assay and 3% gel electrophoresis and were then compared to those of the nested PCR test. The experiments were conducted in triplicate.

### 2.8. Evaluation of the LAMP-LFD Assay Using Hydatid Cyst Samples

Sixty-two hydatid cysts, 33 from liver samples, 27 from lung samples, and 2 from spleen samples collected from cattle slaughtered at Juja Abattoir, Juja Town, Kenya, were used. The samples were microscopically examined to determine the fertility rate [28]. Genomic DNA was extracted from all samples using the alkaline lysis rapid extraction method [29]. Briefly, a small piece of parasite tissue or protoscolices was crushed using a glass pestle with 0.02 M NaOH and boiled at 99°C for 10  minutes. Three microliters of the liquid phase was used directly as the DNA template. All samples were subjected to LAMP-LFD and nested PCR to compare their sensitivity.

### 2.9. Statistical Data Analysis

All data were entered into a Microsoft Excel spreadsheet (Microsoft 2007, USA). Contingency tables were developed, and diagnostic test performance of LAMP-LFD was calculated with 95% confidence intervals for sensitivity and specificity of LAMP-LFD test compared to nested PCR. A Kappa test was used to measure the degree of agreement between the two tests. All statistical analyses of data were carried out with MedCalc® (version 9.6.2.0). GraphPad Prism® software version 7.04 (GraphPad, USA) was used for graph generation. A *P* value <0.05 was considered statistically significant.

## 3. Results

### 3.1. Optimization of the LAMP Conditions

Successful amplification of the target gene (NADH-1 gene) confirmed the specificity of the primers for *E. granulosus.* The optimum temperature for the LAMP reaction was 63°C, and the optimal time was 40 minutes. The MgSO_4_ concentration of 6 mM and 0.4 mM of dNTP mix was optimum for the LAMP assays.

### 3.2. Detection of the LAMP Products

**Figure 2:**
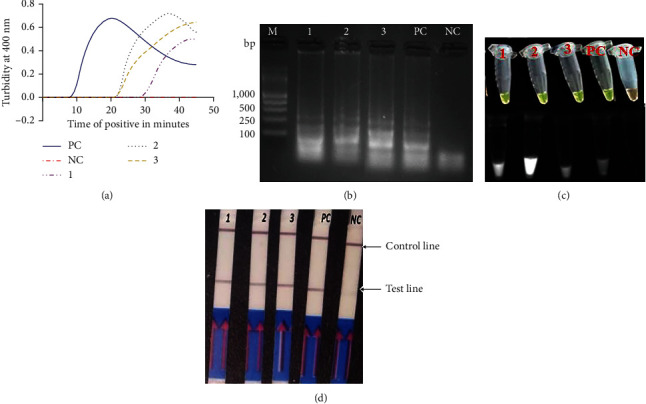
Detection of the LAMP products; the reaction was conducted at 63°C for 40 minutes. (a) Determination of the LAMP turbidity by a real-time turbidimeter. (b) 3% agarose gel electrophoresis of nested PCR products. (c) Visual detection using SYBR Green 1 dye under visible light and UV light. (d) LAMP-LFD detection: CL and TL indicate control line and test line, respectively. Lines 1–3 represent positive samples; PC is positive control and NC is negative control. Lane M: 1 kb DNA molecular marker.

Following the optimization of LAMP reaction conditions, the LAMP amplification was first monitored through the analysis of amplification curves on the Loopamp LA-500 real-time turbidimeter at 400 nm absorbance as shown in Figure 2(a). On agarose gel electrophoresis, the positive samples for *E. granulosus* showed a typical ladder-like pattern (Figure 2(b)), while visual detection with SYBR Green 1 dye showed positive reactions turning green under visible light and negative samples remained light orange. Under UV light, notable fluoresced-bright colors indicated positive reactions with negative samples remaining dark (Figure 2(c)). The LAMP end product was analyzed on lateral flow strips, and the expected two visible bands on the LFD strips consisted of test line (TL) and control line (CL) for positive samples, and one band of control line (CL) was observed for negative samples (Figure 2(d)).

### 3.3. Nested PCR Detection

**Figure 3:**
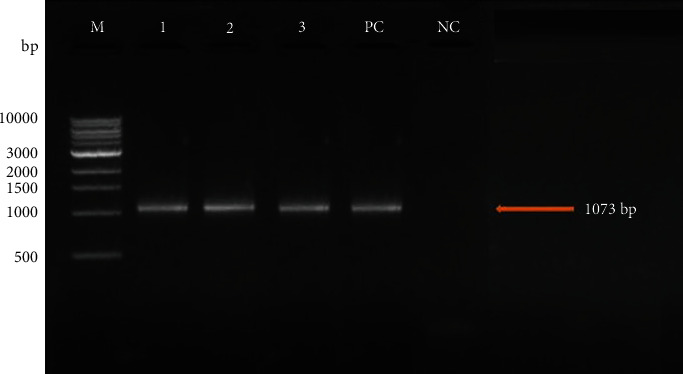
1.5% gel electrophoresis showing detection of nested PCR products (1073 bp) of the NADH-1 gene of *E. granulosus*. Lane M: 1 kb molecular marker; lanes 1–3 are positive samples; lines PC and NC are positive control and negative control, respectively.

The nested PCR assay was employed to amplify the NADH-1 gene of *E. granulosus* using external primers for the first reaction and internal primers for the second reaction. As shown in Figure 3, nested PCR amplification products were detected on a 1.5% gel electrophoresis showing 1073 bp products.

### 3.4. Analytical Sensitivity and Specificity of LAMP-LFD and Nested PCR Assays

**Figure 4:**
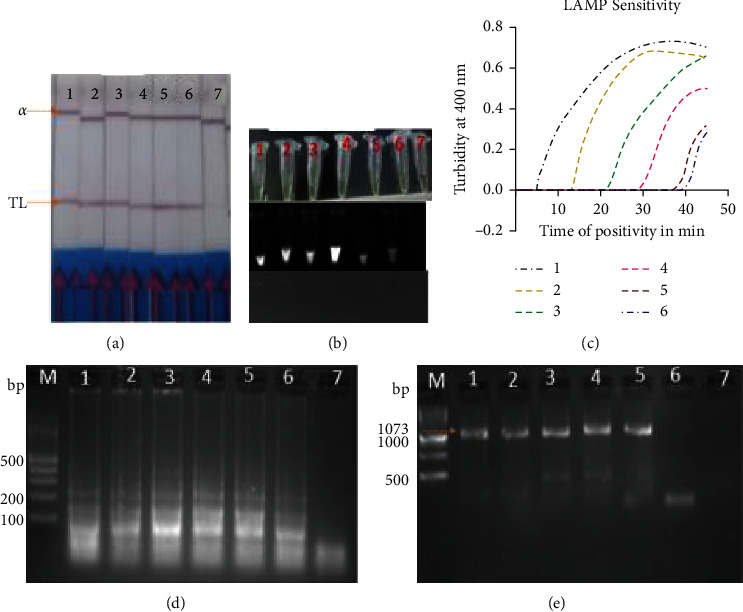
Analytical sensitivity of LAMP and nested PCR products using 10-fold dilutions of *E. granulosus* DNA at 63°C for 40 minutes. (a) LAMP products detected by LFD strips; CL indicates control line and TL indicates test line for positive samples. (b) Visual detection of LAMP products using SYBR Green 1 dye under visible (green) and UV light (bright fluorescence), respectively. (c) Real-time LAMP turbidity monitored by a Loopamp real-time turbidimeter at 400 nm. The amplification curve graph was generated by GraphPad Prism software version 8.00. (d) 3% agarose gel electrophoresis of LAMP products. (e) Nested PCR products analysis on 1.5% agarose gel; M: DNA molecular marker; N: no template control; 1–7: 10-fold serial dilution of *E. granulosus* DNA from 10^−1^ to 10^−7^ (1 ng to 1 fg).

The analytical sensitivity of the LAMP-LFD assay was evaluated through 10-fold dilution of the *E. granulosus* DNA template (1 ng, 100 pg, 10 pg, 1 pg, 100 fg, 10 fg, and 1 fg) and showed a sensitivity level of 10^−6^ (10 fg/*μ*L) (Figure 4). For comparison purposes, the results of the nested PCR assay for the same concentration using outer primer and inner primer sets displayed no bands. With regard to these findings, the limit of LAMP-LFD detection was at least 10 times higher than the gold standard nested PCR (Figure 4).

**Figure 5:**
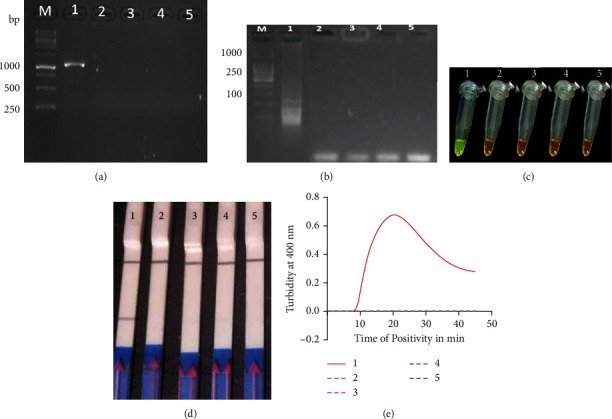
Specificity test. Nested PCR products analysis on 1.5% agarose gel (a); LAMP products analysis on 3% agarose gel (b); SYBR green visual detection (c); LAMP-LFD test (d); real-time turbidity monitoring (e). CL and TL are control and test lines, respectively. Lane M: DNA molecular marker; tube 1 represents *E granulosus* DNA template; tubes 2, 3, and 4 represent DNA templates from *T. saginata, T. solium,* and *F. hepatica,* respectively, while tube 5 represents the no template control.

For specificity assays, nested PCR and LAMP-LFD assays showed positive results with *E. granulosus* DNA with no cross-reactivity with DNA from *T. saginata, T. solium,* and *F. hepatica* (Figure 5).

### 3.5. Evaluation of Diagnostic Performance of the LAMP-LFD Assays

Microscopic examination revealed that 48 cysts (77.4%) were fertile (positive with protoscolices), while 14 cysts (22.6%) were infertile.

The diagnostic sensitivity assays indicated 59/62 (95.2%, 95% CI 0.87–0.98) and 53/62 (85.5%, 95% CI 0.75–0.92) as positive by LAMP-LFD and nested PCR, respectively (Table 2). While both methods positively detected all the fertile cysts, only 11 infertile cysts were detected by LAMP-LFD (78.6%) and 5 (35.7%) by nested PCR. Kappa analysis showed moderate agreement (Kappa = 0.46) for the two tests.

## 4. Discussion

In this study, a LAMP-LFD was successfully developed and evaluated for the detection of *E. granulosus* infection in hydatid cyst samples from slaughtered livestock. LAMP is a single-tube test for DNA amplification and a low-cost alternative to PCR methods [17]. Due to its advantages of high sensitivity, specificity, and applicability in endemic areas with limited diagnostic resources, LAMP has gained a lot of momentum in diagnosing many pathogenic microorganisms in humans, animals, and plants [24]. In contrast to PCR and nested PCR, the detection time of LAMP is less than 1 hour because the target gene is amplified under isothermal conditions [30].

The currently available LAMP detection methods which include real-time turbidimetry (RT-LAMP), gel electrophoresis, and colorimetric indicators are laborious and time-consuming and require sophisticated equipment, and the specificity of colorimetric dyes is low and can bind any double-stranded DNA including primer dimers which may lead to false positives [23, 25]. The LAMP-LFD provides a faster naked-eye visualization of the result and high specificity due to the use of a specific hybridization probe that binds to a distinct sequence of the target gene [31]. The LAMP test was developed by targeting the NADH-1 gene of *E. granulosus* mitochondrial DNA. Under optimal conditions, the LAMP-LFD protocol was completed within 40 minutes at 63°C, with easy naked-eye detection of results within 5 minutes. The overall time spent completing the test was faster than that previously reported [14, 21]. The specific FITC-labeled probe designed and used in this study to detect LAMP products using LFD confirmed superior specificity in the detection of LAMP reaction products. The detection limits were 10 fg of genomic DNA, being more sensitive than previously reported levels of 100 fg [21, 22, 27], and less than the 1 fg for the real-time based LAMP adapted in [15]. Regarding the PCR sensitivity test, the lowest detection of the nested PCR was 100 fg, which was at least 100 times more than conventional PCR.

According to the specificity assays, LAMP-LFD detected only *E. granulosus* DNA with no cross-reactivity. The developed LAMP-LFD assay showed increased sensitivity (95.16%) and moderate agreement compared with that of the gold standard, nested PCR for hydatidosis detection. There was 100% consistency between the results of the LAMP-LFD assay and nested PCR, with LAMP-LFD being more sensitive than nested PCR for the infertile cysts. This could be due to the limitations of PCR to detect minute amounts of DNA.

A similar superior sensitivity of the LAMP-LFD assay over nested PCR has been reported in the diagnosis of *Cryptosporidium* species in Kenya [24]. Other studies have also recorded the high sensitivity of the LAMP assay over nested PCR for the detection of *Trypanosoma gambiense* [32] and Pneumocystis jirovecii in immunocompromised patients [33].

Based on the results of this study, the application of LFD strips for the analysis of LAMP amplicons could reduce the total analysis time and complications associated with usual detection and gel electrophoresis. In reference to the results of this study, the high sensitivity and specificity with relatively short analysis time and the use of relatively nonsophisticated and inexpensive equipment were key advantages of the LAMP-LFD test. This is the first study proposing the use of the LAMP-LFD assay for the detection of *E. granulosus*, so far, whether in intermediate or definitive hosts.

## 5. Conclusion

The present study demonstrated that the LAMP method combined with lateral flow detection has high sensitivity and specificity for the diagnosis of *E. granulosus* infection in livestock. Compared to nested PCR, the LAMP-LFD has advantages which include simplicity, efficiency, sensitivity, and specificity. The LAMP technique does not require specific, expensive, and sophisticated equipment, and a simple heat block or water bath is sufficient to provide a constant temperature for reactions that require less than one hour. Another useful feature of LAMP LFD is that positive and negative samples can be discriminated by observing the lateral flow strips using naked eyes. Therefore, the LAMP-LFD method is a promising assay for wide application and rapid detection of *E. granulosus* infection and could be useful for monitoring CE in livestock.

## Figures and Tables

**Table 1:** LAMP primers and probe used in this study.

Primer name	Sequences (5ʹ-3ʹ)
F3	*TGGTGGTTTTGTTTATCAGG*
B3	*CGACTTTGGAAGTAAAAACACTT*
FIP	*CTCCCCTAAAACAAAAAAGGCAATTCAGGTGGTTGTTTTTGGGT*
BIP	*TTGGGCTATTCTCAGTCTCGTATAGATCAGCAAACCTCTGC*
LF	*CAAACCAAAAACTCCAGAGACT*
LB	*AAGGGCCCTAACAAGGTTGG*
Probe	*TTGTTTTAGGGGAGCGTAAGT*

**Table 2:** Comparison between LAMP-LFD and nested PCR tests for detection of *E*. *granulosus* from fertile and infertile cysts.

Type of samples	LAMP-LFD results	Nested PCR results
Fertile cyst (*N* = 48)	48 (100%)	48 (100%)
Infertile cysts (*N* = 14)	11 (78.6%)	5 (45.5%)
Overall detection	59 (95.16%)	53 (85.48%)

## Data Availability

The data used to support the findings of this study are available from the corresponding author upon request.

## Abbreviations

BP: Base pair
CI: Confidence interval
LAMP: Loop-mediated isothermal amplification
LFD: Lateral flow dipstick
NAD: Nicotinamide adenine dinucleotide
PCR: Polymerase chain reaction
POC: Point of care
RT-PCR: Real-time polymerase chain reaction
F3/B3: Forward/backward outer primer
FIP/BIP: Forward/backward inner primer
LF/LB: Forward/backward loop primer.
